# Target Site of Prepulse Inhibition of the Trigeminal Blink Reflex in Humans

**DOI:** 10.1523/JNEUROSCI.1468-22.2022

**Published:** 2023-01-11

**Authors:** Koji Inui, Yasushi Itoh, Borgil Bayasgalan, Megumi Shingaki, Tomoya Taniguchi, Eishi Motomura, Tetsuo Kida

**Affiliations:** ^1^Department of Functioning and Disability, Institute for Developmental Research, Aichi Developmental Disability Center, Kasugai 480-0392, Japan; ^2^Department of Integrative Physiology, National Institute for Physiological Sciences, Okazaki 444-8585, Japan; ^3^Department of Anesthesiology, Nagoya University Graduate School of Medicine, Nagoya 466-8550, Japan; ^4^Department of Neuropsychiatry, Mie University Graduate School of Medicine, Tsu 514-8507, Japan

**Keywords:** electromyography, mechanomyography, R1, R2, R3, sensorimotor gating

## Abstract

Despite the clinical significance of prepulse inhibition (PPI), the mechanisms are not well understood. Herein, we present our investigation of PPI in the R1 component of electrically induced blink reflexes. The effect of a prepulse was explored with varying prepulse test intervals (PTIs) of 20–600 ms in 4 females and 12 males. Prepulse–test combinations included the following: stimulation of the supraorbital nerve (SON)–SON [Experiment (Exp) 1], sound–sound (Exp 2), the axon of the facial nerve–SON (Exp 3), sound–SON (Exp 4), and SON–SON with a long trial–trial interval (Exp 5). Results showed that (1) leading weak SON stimulation reduced SON-induced ipsilateral R1 with a maximum effect at a PTI of 140 ms, (2) the sound–sound paradigm resulted in a U-shaped inhibition time course of the auditory startle reflex (ASR) peaking at 140 ms PTI, (3) facial nerve stimulation showed only a weak effect on R1, (4) a weak sound prepulse facilitated R1 but strongly inhibited SON-induced late blink reflexes (LateRs) with a similar U-shaped curve, and (5) LateR in Exp 5 was almost completely absent at PTIs >80 ms. These results indicate that the principal sensory nucleus is responsible for R1 PPI. Inhibition of ASR or LateR occurs at a point in the startle reflex circuit where auditory and somatosensory signals converge. Although the two inhibitions are different in location, their similar time courses suggest similar neural mechanisms. As R1 has a simple circuit and is stable, R1 PPI helps to clarify PPI mechanisms.

**SIGNIFICANCE STATEMENT** Prepulse inhibition (PPI) is a phenomenon in which the startle response induced by a startle stimulus is suppressed by a preceding nonstartle stimulus. This study demonstrated that the R1 component of the trigeminal blink reflex shows clear PPI despite R1 generation within a circuit consisting of the trigeminal and facial nuclei, without startle reflex circuit involvement. Thus, PPI is not specific to the startle reflex. In addition, PPI of R1, the auditory startle reflex, and the trigeminal late blink reflex showed similar time courses in response to the prepulse test interval, suggesting similar mechanisms regardless of inhibition site. R1 PPI, in conjunction with other paradigms with different prepulse–test combinations, would increase understanding of the underlying mechanisms.

## Introduction

Although inhibitory control of excitatory pyramidal neurons is critical for brain function (Hennequin et al., 2017; Maffei, 2017), inhibition measurement is generally difficult. One reason for this is the near impossibility of noninvasively observing IPSPs in humans, which are important in excitatory synapse output determination. One indirect method of inhibition observation is paired-pulse suppression, in which two identical sensory stimuli are presented successively, evaluating the change in response of the second stimulus to the first (Kimura, 1973; Pellegrini and Evinger, 1995). Through this, one can evaluate the circuit excitability or excitation/inhibition balance (Schicatano et al., 2000). For example, one experiment is two successive supraorbital nerve (SON) stimulations, while varying the interval between the two stimuli, then comparing the evoked blink response magnitude between the two stimuli. In normal subjects, the blink reflex is inhibited at certain intervals, whereas patients with Parkinsonism show decreased inhibition, thus implicating reflex circuit excitability changes in this disease (Kimura, 1973). Another well known example is P50 gating, in which the positive deflection amplitude at ∼50 ms in auditory evoked potentials (EPs) is compared between two successive click sounds spaced by 500 ms (Adler et al., 1982).

When the first stimulus is weak and does not reliably elicit a response, the paradigm is called prepulse inhibition (PPI; Graham, 1975); this describes a weak leading stimulus (prepulse) that reduces the response to a subsequent stronger stimulus (test). Usually, startle reflexes, such as those elicited by loud sounds, are used as the test response. PPI is considered to reflect a sensorimotor inhibition process that prevents disruption in processing the first stimulus by interference from the second, stronger sensory input, thus maintaining attention on the initially detected aspect of the environment (Braff et al., 2001). One attractive feature of PPI is the commonality across mammals, which advanced translational studies, including those using animal models. It is particularly important in schizophrenia as both patients and animal models show PPI deficits (Swerdlow et al., 2008). Thus, knowledge of the PPI mechanism is essential to understanding the neural basis of cognition and reflex, as well as the pathophysiology of certain diseases (Fendt et al., 2001; Swerdlow et al., 2001).

Along with these implications, PPI may reflect a fundamental inhibitory process, not just limited to the startle reflex, and thus provide insight into underlying brain inhibitory mechanisms. Auditory-evoked cortical responses are known to be inhibited by a preceding weak prepulse (Inui et al., 2012, 2016), a process involving GABAergic mechanisms (Inui et al., 2018). Similar findings are reported for somatosensory-evoked cortical responses (Nakagawa et al., 2014), supporting the ubiquity of PPI. One issue with standard PPI paradigms is that the startle reflex circuit is not fully elucidated, leaving questions of neural inhibition mechanisms unsolved (Swerdlow et al., 2001; Valls-Sole, 2012), for example, where and how signals evoked by startle stimuli are inhibited.

In this study, we investigated the PPI of an early component of the electrically induced blink reflex, which is an oligosynaptic reflex via the principal nucleus of the trigeminal nerve and facial nucleus. As inhibition can occur at each step within the circuit, increasing circuit complexity increases inhibition mechanism complexity. With this in mind, we aimed to determine the site of inhibition using five experiments.

## Materials and Methods

This study was approved in advance by the Ethics Committee of Aichi Developmental Disability Center (Kasugai, Japan; approval no. R0110) and was conducted in accordance with the Declaration of Helsinki. Written consent was obtained from all subjects. The study was performed in 16 healthy volunteers (4 females and 12 males; mean age, 34.3 ± 12.6 years; age range, 20–57 years). The sample size was determined by a priori analysis using a repeated-measures *F* test within-factors design (G*Power 3.1; *n* = 15 with group number = 1, measurement number = 9, effect size = 0.25, α error probability = 0.05, power = 0.8, correlation among repeated measures = 0.5; and *n* = 16 when measurement number = 8 for the main effects). None of the subjects were treated for neurologic or mental diseases or substance abuse in the last 2 years.

### Experimental design

Four experiments (Exps; 1–4) were conducted in 16 subjects; Exp 1 and Exp 2 were conducted in 1 d, and then Exp 3 and Exp 4 were conducted on another day approximately a week later. Finally, Exp 5 was conducted with 10 subjects. In all experiments, there were 10 stimulus conditions including test alone, prepulse alone, test plus prepulse with prepulse test intervals (PTIs) of 20, 50, 80, 110, 140, 200, 400, and 600 ms. The recording of stimulus conditions was divided into three blocks with fixed combinations: test alone; prepulse alone; test plus prepulse with PTIs of 20 and 50 ms in Block 1; test alone plus PTIs of 80, 110, and 140 ms in Block 2; and test alone plus PTIs of 200, 400, and 600 ms in Block 3. Block order was randomized across subjects.

### Electrical and auditory stimulation

To elicit blink reflexes, the right SON was stimulated with a square wave pulse of 0.5 ms using two disposable Ag/AgCl gel electrodes 10 mm in diameter (Biorode SDC-H, Vyaire Medical) placed on the supraorbital foramen and ∼3 cm above it, respectively.

The slightly longer than usual stimulus duration was to reduce the stimulus current and voltage. The R1 threshold was defined as a current at which R1 was elicited in 50% of stimulations, and was determined by an up-down procedure before the experiment in each subject. The current intensity was 1.5 times the threshold for the test stimulus and 0.9 times for the prepulse. In Experiment 3, the axon of the right facial nerve was electrically stimulated with a 0.5 ms square pulse. Two electrodes were placed on an area just anterior to the ear lobe as cathode and the mastoid as anode, respectively. The threshold was determined similarly and stimulation at 0.9 times the M-wave threshold was used.

In Exp 2 and Exp 4, the continuous white noise at 70 dB was presented binaurally by headphones. The test sound stimulus was 40 ms white noise of 115 dB, and the prepulse was 20 ms at 85 dB. Sounds were created by a personal computer (Windows XP, 32 bit) equipped with a sound card (Sound Blaster 5/Rx, CREATIVE) and a headphone amplifier (AT-HA21, Audio-Technica).

### Recording and analysis of the blink reflex

**Figure 1.**
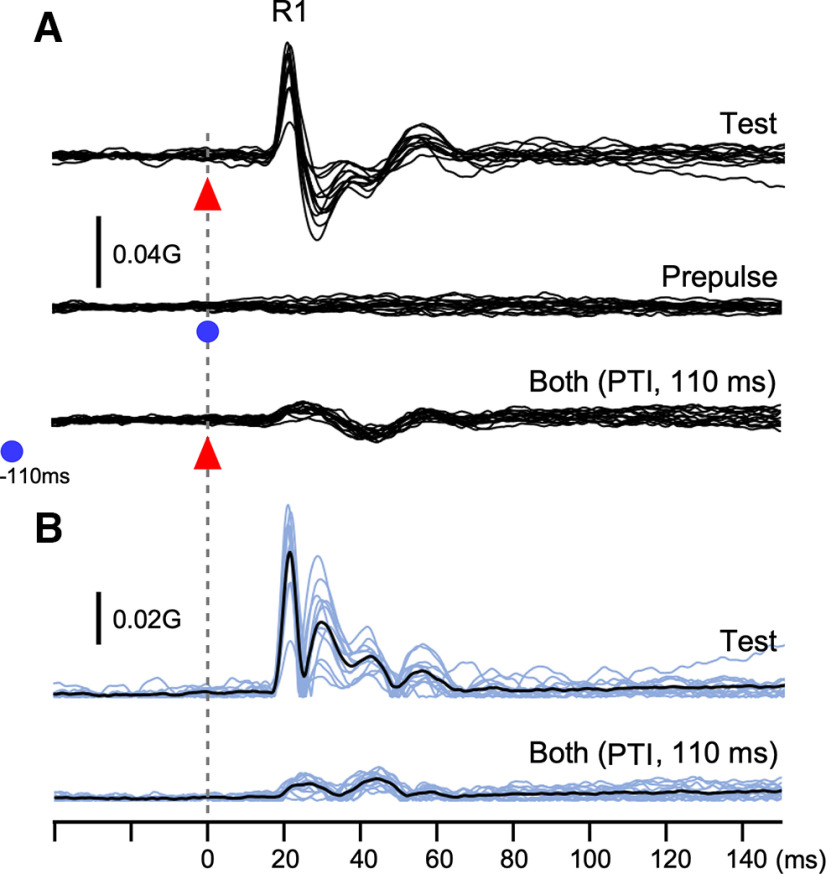
R1 component of the electrically induced blink reflex recorded by an accelerometer. An example of R1 elicited by electrical stimulation of the supraorbital nerve. ***A***, Superimposed 15 waveforms elicited by the test stimulus alone, prepulse alone, and test plus prepulse with a PTI of 110 ms. Filled triangles and circles indicate the test stimulus onset and prepulse onset, respectively. ***B***, Full rectified waveforms of the 15 sweeps (blue) and their average (black).

Subjects were seated in a chair and instructed to gaze at a fixed point 1.5 m in front of them with their eyes open. Blink reflexes were recorded from the orbicularis muscles using a single-axis accelerometer (8 × 8 × 4 mm; MPS110, Medi Sens) placed on the central part of the lower eyelid. The analog filter was set at 1–250 Hz. Signals were amplified and stored in an electromyography (EMG)/EP measuring system (MEB-2300, Nihon Kohden) at a sampling rate of 10,000 Hz. The analysis window was 60 ms before to 240 ms after stimulation in Exp 2, and 40 ms before to 160 ms after the onset of stimulation in other experiments. Each response was fully rectified and averaged across a block. The prestimulus baseline was subtracted, and then the area under the curves (AUCs) at 15–40 ms was calculated as the R1 response. For the auditory startle reflex in Exp 2, AUC at 50–120 ms was calculated. Because SON stimulation with a long trial–trial interval elicits ipsilateral R1 followed by bilateral later components, R2 and sometimes R3, the later components were evaluated using an AUC at 60–100 ms in Exp 4 and Exp 5. Although the R2 component starts at ∼30 ms with a duration of ∼30 ms on EMGs (Kugelberg, 1952; Aramideh and Ongerboer de Visser, 2002), we selected a 60–100 ms analysis period because eyelid movement because of the R1 component appears to continue up to ∼60 ms [Fig. 1 (see also Fig. 3)]. The R2 and R3 components were not distinguishable in the present study, and therefore, the later component analyzed at this latency was referred to as the late blink reflex (LateR).

### Experimental conditions

#### Experiment 1.

Both the test stimulus and prepulse were SON stimulation. The stimulus frequency was 1 Hz for Blocks 1–3, and 0.7 Hz for Block 4. The latter was used to avoid shorter interstimulus intervals than the PTI. Each stimulus was delivered 16 times in one run, and two runs were performed for each stimulus condition in random order. In all experiments, the first trial of a run was excluded from averaging.

#### Experiment 2.

The test and prepulse stimulus were an abrupt increase in sound pressure from the background white noise of 70 dB, by 45 dB for 40 ms and 15 dB for 20 ms, respectively. The stimulus was delivered every 14–16 s. For all blocks, five responses were recorded for each stimulus in a run, and two runs were performed in random order. Unlike the other four experiments, the four stimuli in a block were randomly presented.

#### Experiment 3.

The test stimulus was SON stimulation, and the prepulse was stimulation of the facial nerve. Other procedures were as per Exp 1.

#### Experiment 4.

The test stimulus was SON stimulation, and the prepulse was a sound pressure increase for 20 ms by 15 dB from the background noise of 70 dB. The stimuli were presented every 14–16 s. As long-interval stimulation of SON causes LateR in addition to R1, the effects of the prepulse on both components were analyzed.

#### Experiment 5.

As only 10 of 16 subjects were available for testing, SON stimulation was used for both the test and prepulse as per Exp 1 with these subjects. The procedures differed from Exp 1 in that the trial–trial interval was 14–16 s, and the averaging was five epochs in a block. Like in Exp 4, both the R1 and LateR components were analyzed.

### Statistical analyses

The degree of inhibition was calculated as the percentage of the response amplitude of a test plus the prepulse condition, relative to the test alone condition of the same block (%Amplitude). Significant differences in the degree of inhibition among eight PTI conditions were analyzed using one-way repeated-measures ANOVA. To compare differences between pairs, *post hoc* multiple comparisons were performed using Bonferroni-adjusted *t* tests. Significance of the prepulse effect on each test plus the prepulse response was assessed using 95% confidence intervals (CIs) of the %Amplitude, with CIs not overlapping 100 determined to be significant. For comparisons of the degree of R1 inhibition between Exp 1 and Exp 5 as well as the degree of LateR inhibition between Exp 4 and Exp 5, differences in %Amplitude at each PTI were analyzed using paired *t* tests. The statistical significance was set at *p* values <0.05. For statistical analyses, SPSS version 24 was used. Data were expressed as the mean ± SD.

## Results

**Figure 2.**
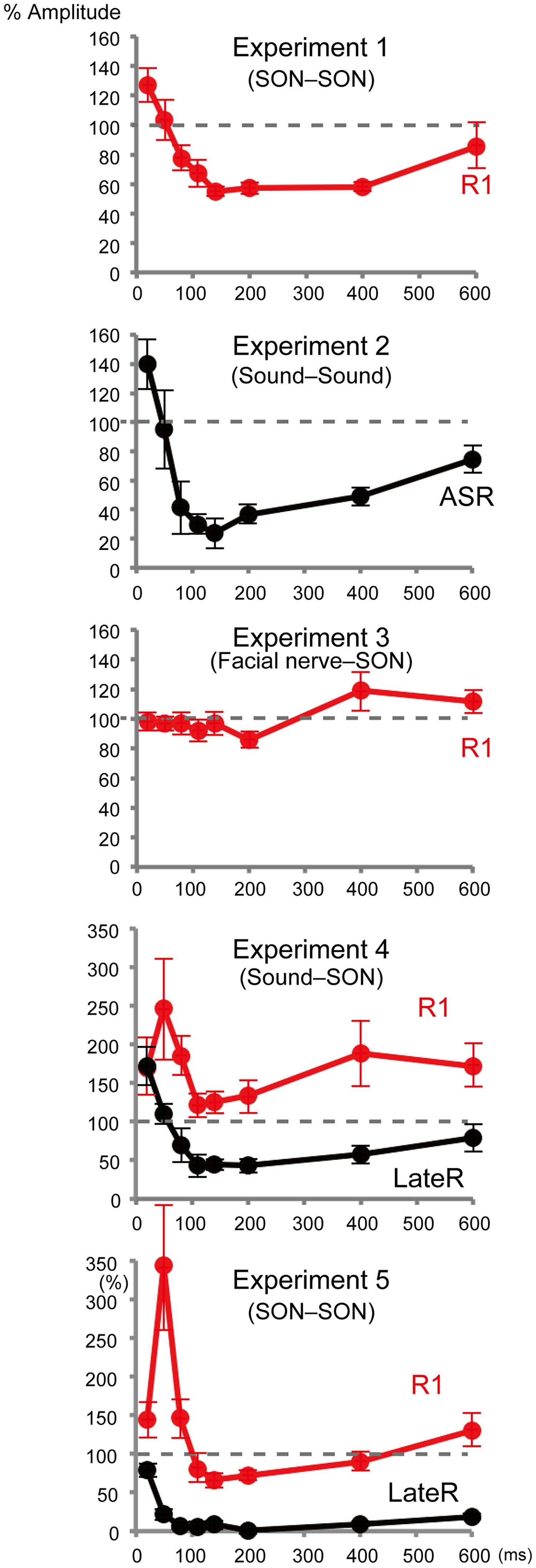
The time course of prepulse inhibition as a function of PTI. The %Amplitude indicates the percentage response amplitude of each condition relative to the response to the test stimulus alone. Vertical bars indicate ±SEs.

**Figure 3.**
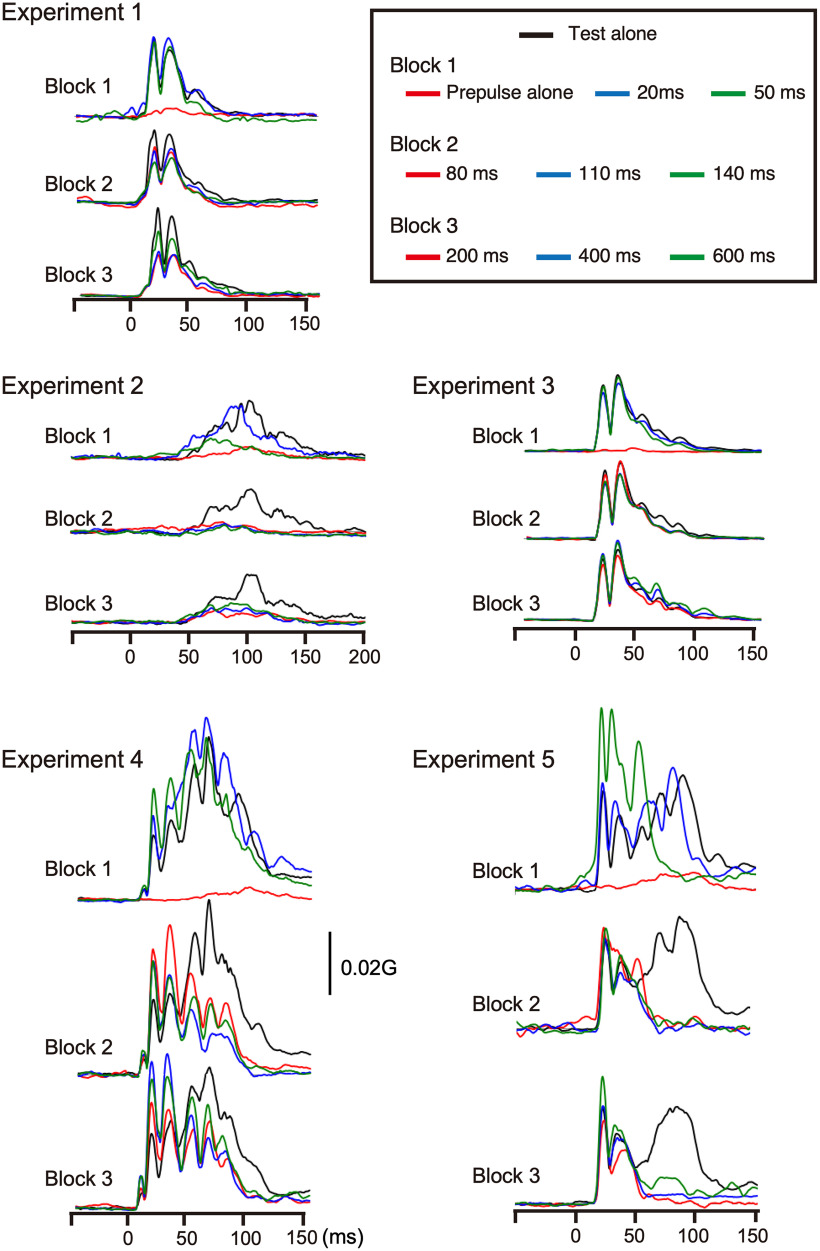
Grand-averaged rectified waveforms. Grand-averaged waveforms of all five experiments are shown. In each experiment, there were three blocks, with four stimuli in each.

Electrical stimulation of the SON elicited a biphasic R1 response ipsilaterally with the first signal peak at 20–25 ms (Fig. 1*A*), which is delayed by 10–15 ms from the corresponding peak in the EMG. The time gap closely matched the 11–12 ms delay between EMG R1 and eyelid closure as reported by Evinger et al. (1991). In Exp 1, effects because of a weak prepulse (0.9 times the R1 threshold) of SON stimulation on R1 elicited by test SON stimulation (1.5 times the threshold) were examined. The mean response amplitude for all conditions is listed in Table 1. Figure 2*A* shows the percentage of R1 amplitude relative to the response elicited by the test stimulus alone (%Amplitude) at PTIs of 20–600 ms. The %Amplitude for all conditions is listed in Table 2. Asterisks in Table 2 show conditions in which the prepulse significantly affected the test response as judged by CIs. The %Amplitude was significantly different among eight PTI conditions (ANOVA: *F*_(7,105)_ = 9.01, *p* = 1.1 × 10^−8^, partial η^2^ = 0.38) and followed a U-shaped curve, with peak inhibition at a PTI of 140 ms. *Post hoc* tests revealed four pairs with a significant difference between 20 and 140 ms, 20 and 200 ms, 20 and 400 ms, and 400 and 600 ms. Grand-averaged rectified waveforms are shown in Figure 3. In Exp 2, similar effects were examined using a weak (85 dB) prepulse sound followed by the test sound (115 dB). The PTI significantly affected the %Amplitude of the auditory startle reflex (ASR; *F*_(7,105)_ = 7.48, *p* = 2.8 × 10^−7^, partial η^2^ = 0.33) showing a U-shaped curve similar to Exp 1 (Fig. 2*B*). Results of *post hoc* tests showed five pairs with a significant difference between 20 and 80–400 ms.

**Table 1.** The mean amplitude (AUC) of the response

		Exp 1	Exp 2	Exp 3	Exp 4		Exp 5	
					R1	LateR	R1	LateR
Block1	Test alone	1.7 (1.5)	2.2 (2.9)	1.9 (2.6)	1.7 (1.5)	6.5 (6.9)	2.0 (1.6)	4.4 (2.4)
	Prepulse alone	0.2 (0.4)	0.9 (2.0)	0.0 (0.1)	0.0 (0.1)	0.4 (0.6)	0.1 (0.2)	0.6 (0.4)
	20 ms	2.1 (2.1)	1.8 (1.7)	1.8 (2.4)	2.2 (1.7)	8.3 (7.2)	2.4 (1.6)	3.3 (2.3)
	50 ms	1.7 (1.5)	0.8 (0.9)	1.8 (2.5)	2.9 (2.3)	6.2 (6.6)	4.6 (2.3)	1.0 (1.0)
Block2	Test alone	1.8 (1.7)	1.7 (2.1)	1.9 (3.1)	1.9 (1.5)	6.7 (7.2)	2.1 (1.4)	4.4 (2.7)
	80 ms	1.2 (1.1)	0.4 (0.4)	1.9 (3.0)	3.4 (3.2)	3.4 (3.6)	2.7 (1.5)	0.6 (0.9)
	110 ms	1.1 (1.1)	0.2 (0.5)	1.7 (2.4)	2.5 (2.6)	2.0 (3.0)	1.8 (1.5)	0.4 (0.8)
	140 ms	1.0 (1.2)	0.2 (0.3)	1.7 (2.4)	2.4 (2.3)	3.0 (3.8)	1.6 (1.3)	0.5 (0.8)
Block3	Test alone	2.0 (2.0)	1.8 (2.0)	1.7 (2.6)	1.8 (1.6)	6.2 (5.8)	2.2 (1.7)	4.4 (2.7)
	200 ms	1.0 (1.1)	0.6 (0.8)	1.6 (2.5)	2.4 (2.6)	3.2 (4.4)	1.6 (1.3)	0.1 (0.2)
	400 ms	1.1 (1.0)	0.8 (1.0)	1.9 (2.7)	3.4 (4.7)	3.1 (3.3)	1.9 (1.5)	0.5 (0.6)
	600 ms	1.5 (1.4)	1.0 (1.1)	1.9 (2.7)	3.1 (3.7)	4.0 (4.7)	2.6 (1.7)	1.1 (1.2)

**Table 2.** Percentage amplitude relative to the test alone response

PTI	Exp 1	Exp 2	Exp3	Exp 4		Exp 5	
				R1	LateR	R1	LateR
20 ms	127 (45)*	140 (68)*	98 (24)	170 (153)	172 (102)*	144 (75)	79 (28)*
50 ms	103 (54)	95 (107)	97 (18)	247 (269)	110 (53)	345 (263)*	22 (22)*
80 ms	77 (34)*	41 (70)*	97 (30)	285 (104)	70 (90)	146 (82)	6 (14)*
110 ms	67 (36)*	30 (27)*	92 (29)	122 (63)	44 (60)*	80 (61)	6 (17)*
140 ms	55 (25)*	24 (41)*	97 (31)	126 (58)	44 (28)*	67 (30)*	9 (18)*
200 ms	57 (30)*	36 (26)*	86 (22)*	134 (88)	43 (36)*	71 (21)*	1 (2)*
400 ms	58 (24)*	49 (39)*	119 (52)	188 (174)	57 (47)*	89 (40)	9 (10)*
600 ms	85 (37)	75 (61)	112 (31)	171 (116)	80 (73)	130 (70)	18 (12)*

*Conditions in which the prepulse significantly affected the test response as judged by CIs.

In Exp 3, we examined whether such effects occur at the periphery using facial nerve axon stimulation as the prepulse. The PTI significantly affected the %Amplitude (*F*_(7,105)_ = 2.27, *p* = 0.034, partial η^2^ = 0.13). However, *post hoc* tests revealed that only one pair, 200 and 600 ms, showed a significant difference. Exp 4 examined the effects of a weak sound on R1 and LateRs induced by SON stimulation. Unlike Exp 1, in which stimulation was at 1 Hz, SON in Exp 4 was stimulated every 14–16 s to elicit both R1 and later components. The sound prepulse tended to augment R1 throughout the PTIs (*F*_(7,105)_ = 1.90, *p* = 0.08, partial η^2^ = 0.11), while LateR was strongly inhibited (*F*_(7,105)_ = 8.99, *p* = 1.2 × 10^−8^, partial η^2^ = 0.38). Like R1 in Exp 1 and ASR in Exp 2, the LateR amplitude increased at short PTIs, with maximum inhibition at 110–200 ms, gradually recovering at longer PTIs (Figs. 2*D*, 3*D*). *Post hoc* tests showed significant differences for LateR in the following six pairs: between 20 and 110 ms, 20 and 140 ms, 20 and 200 ms, 20 and 400 ms, 50 and 140 ms, and 50 and 200 ms.

Following these results, Exp 5 was conducted in 10 subjects to clarify whether SON stimulation frequency affects R1 inhibition, and whether prepulse–test combination influences the degree of LateR inhibition. Results showed very strong LateR inhibition while only slightly reducing R1 at a few PTIs. As for LateR, the %Amplitude was significantly different among PTIs (*F*_(7,63)_ = 23.6, *p* = 2.2 × 10^−15^, partial η^2^ = 0.72). *Post hoc* testing showed that %Amplitude differences were significant for seven pairs, 20 and 50–600 ms. When the %Amplitude was compared between Exp 4 and Exp 5, inhibition was significantly greater at all PTIs for Exp 5 (paired *t* test, *p* < 0.041), suggesting that a prepulse–test combination of the same stimuli exerted stronger effects. As for R1, despite significantly different %Amplitude among the eight PTI conditions (*F*_(7,63)_ = 7.71, *p* = 1.0 × 10^−6^, partial η^2^ = 0.46), *post hoc* tests showed no pair with a significant difference. As shown in Table 2, significant inhibition was found at 140 and 200 ms PTIs. When the %Amplitude was compared at each PTI between Exp 1 and Exp 5, the mean value was smaller for Exp 1 at all PTIs (Table 2), and the difference was significant at 50, 80, 140, 200, 400, and 600 ms (paired *t* test, *p* < 0.038). As the experimental conditions of Exp 1 and Exp 5 were identical to the exception of the trial–trial interval (1 vs 15 s), the stimulation frequency was a factor to determine the degree of R1 inhibition.

**Figure 4.**
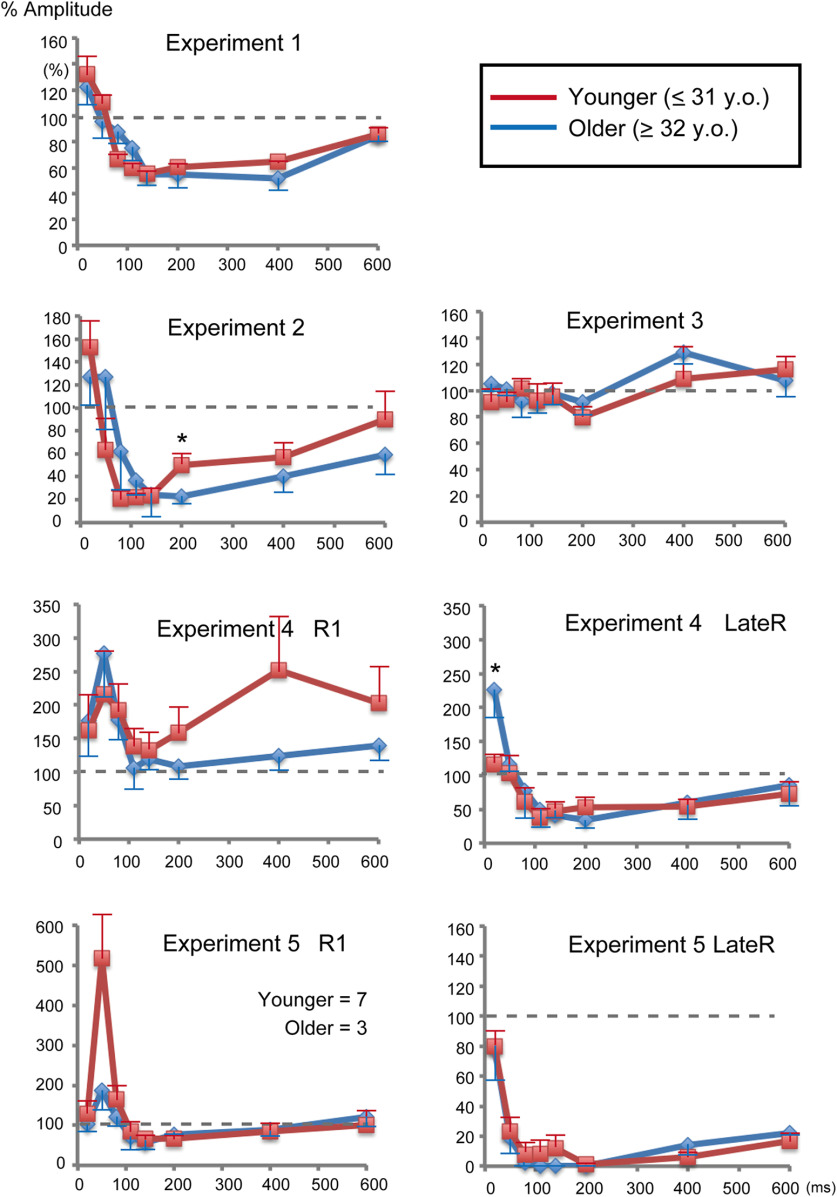
Effects of age on prepulse inhibition. Sixteen subjects were divided into younger (≤31 years of age; *n* = 8) and older (≥32 years of age; *n* = 8) groups to evaluate age effects on the prepulse inhibition at each prepulse test interval. In Experiment 5, there were 7 and 3 subjects in younger and older groups, respectively. Vertical bars indicate 1 SE (younger) or –1 SE (older). Asterisks show a significant difference between the two groups (*t* test, *p* < 0.05 uncorrected for multiple comparisons).

**Figure 5.**
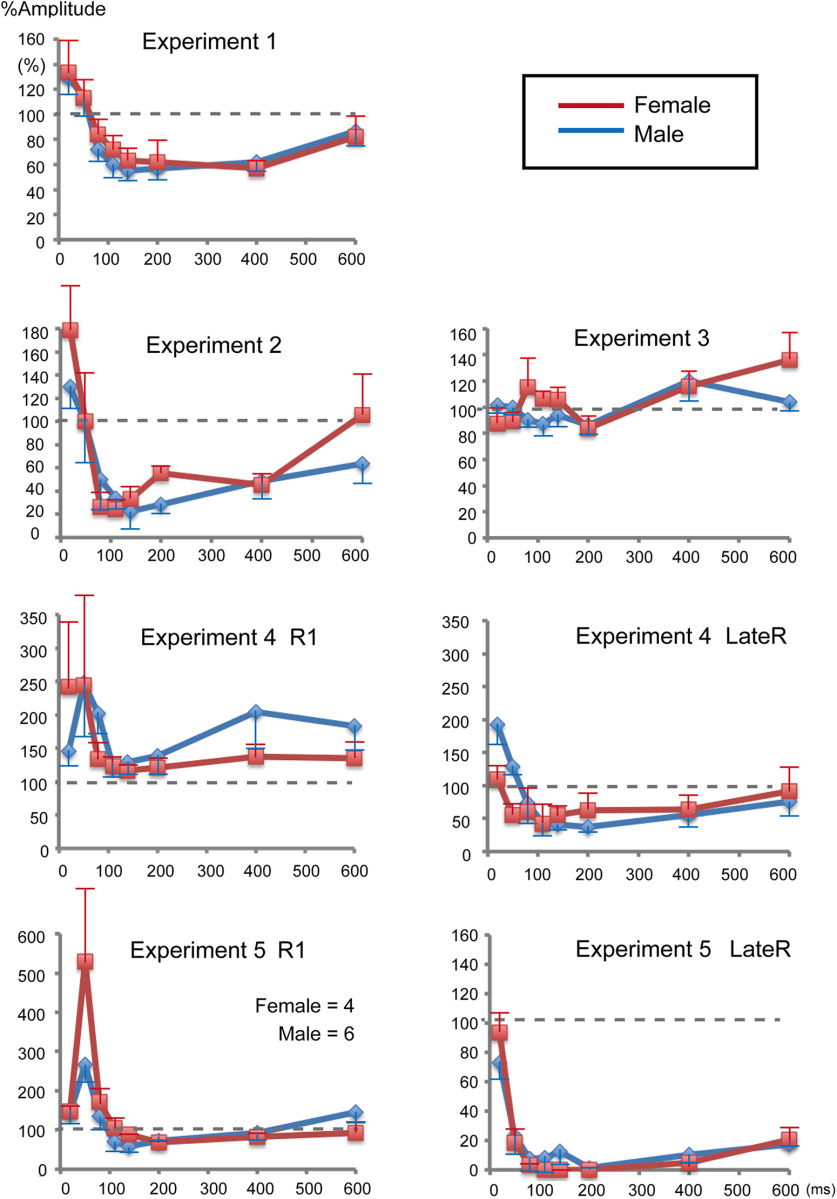
Gender effects on prepulse inhibition. Plots of both genders are shown overlaid. The number of female subjects was four in all five experiments. Bars indicate 1 SE (female) or –1 SE (male).

In this study, influences on the results because of the small sample size, gender bias, and the wide age range of the subjects may exist. To evaluate this, %Amplitude values were compared between younger and older groups, and between males and females. As shown in Figures 4 and 5, such effects appear small, although they cannot be completely ruled out. When the difference between the two groups was analyzed with a *t* test, there were no significant differences between males and females for any PTIs (*p* > 0.094, uncorrected for multiple comparisons). As for the age effect, significant differences were found for the 200 ms PTI of Exp 2 (*p* = 0.031) and the 20 ms PTI of Exp 4 LateR (*p* = 0.026; Fig. 4).

## Discussion

### Target sites of inhibition

The R1 component of electrically induced blink reflexes is an oligosynaptic reflex through the principal sensory nucleus of the trigeminal nerve and facial nucleus (Aramideh et al., 1997; May and Warren, 2021). The modest effect because of preceding facial nerve stimulation indicated that the inhibition site is either or both. As for the possibility of the facial nucleus as the target site, the sound prepulse significantly facilitated R1 in Exp 4, as already shown (Boelhouwer et al., 1991). Sound stimuli with an interval of 14–16 s activate the brainstem auditory areas, auditory cortex, startle reflex circuit, and then facial nucleus, but not the principal sensory nucleus. As facilitation of R1 by the sound prepulse occurred at the facial nucleus, this shows that subthreshold activation of the facial nucleus did not inhibit R1. As those same motor neurons in the facial nucleus contribute to both R1 and later components (Dengler et al., 1982), motor neurons in the facial nucleus appear facilitatory for multiple inputs. The fact that R1 inhibition was weaker for the SON–SON paradigm with a long trial–trial interval (Exp 5) than that in Exp 1 supported this view. Given stimulation with the long trial–trial interval elicits both R1 and later components (R2 and R3), while 1 Hz stimulation only elicits R1 with extremely weak R2, inputs to the facial nucleus should be greater, in total, for Exp 5. If the excitability changes in the facial nucleus are related to R1 inhibition, then the inhibition rate should have been greater in Exp 5, but this was not observed, which instead supports the facilitatory nature of the facial motoneurons. Therefore, these results indicate that the principal nucleus is the target site of R1 prepulse inhibition, which is supported by a paired-pulse study on guinea pigs (Pellegrini and Evinger, 1995).

**Figure 6.**
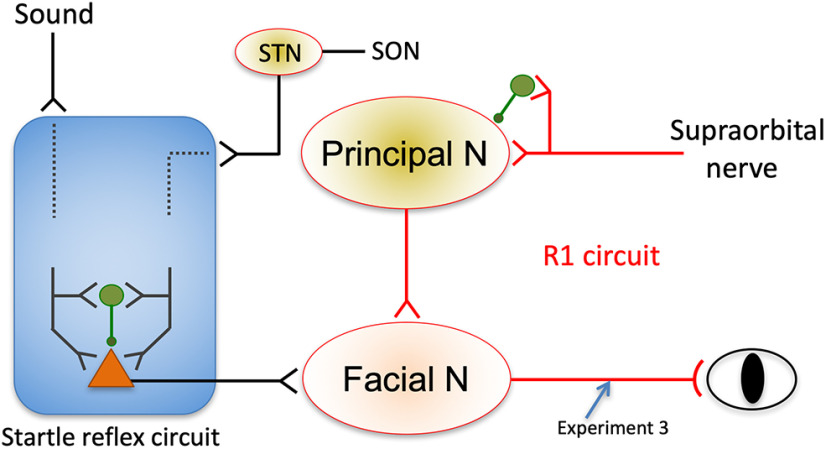
Schematic drawing of the R1 and startle reflex circuits. Red lines indicate the R1 circuit. Green-filled circles indicate an inhibitory interneuron and its synapse on the target neuron. Note that although a simple feedforward type inhibition is shown, various patterns are possible. Principal N, Principal nucleus of the trigeminal nerve; STN, spinal trigeminal nucleus.

The finding that R1 suppression was modest in Exp 5 requires discussion. As the only experimental difference between Exp 1 and Exp 5 was the trial–trial interval, that R1 inhibition was affected should be considered. Previous studies, to the best of our knowledge, have not shown clear PPI of R1 except in one preliminary report (Rossi and Scarpini, 1992). The effect of preceding SON stimulation on SON-induced R1 was reported to be either weak or facilitatory in studies using PPI or paired-pulse paradigms (Kimura, 1973; Powers et al., 1997). For example, one study used a paired-pulse paradigm in which two identical stimuli were delivered to the SON with a trial–trial interval of 30 s, Kimura (1973) reported slight facilitation at a PTI of 60 ms, a slight inhibition at 80–125 ms (∼20% reduction), and gradual recovery at longer PTIs, very similar to our results in Exp 5. As long trial–trial intervals are usually used to observe later blink reflex components, the lack of significant R1 inhibition in previous studies may be because of the stimulation frequency. As the facial nucleus is facilitatory for multiple inputs, any sources activating the facial nucleus should facilitate SON-induced R1. Such sources include circuits for SON-induced LateR and the startle reflex by any sensory modalities (Fig. 6). In this respect, the prominent R1 facilitation at the PTI of 50 ms in Exp 4 and Exp 5 is informative. By considering the onset latency of LateR and ASR (∼30–40 ms) and the time taken to travel from the facial nucleus to the muscle, it will take ∼25–35 ms for the signals elicited by the prepulse to reach the facial nucleus, which are then followed by signals elicited by SON test stimulation with a 50 ms PTI. Under the present experimental conditions, the 50 ms PTI must have been the best to facilitate R1.

ASR, as expected, was strongly inhibited by the weak sound prepulse. As auditory prepulses inhibit auditory-evoked cortical responses (Inui et al., 2016), it is possible that the test response inhibition occurs at more than one site in the auditory pathway. Although the degree of inhibition cannot be easily compared among different sensory systems, one reason for greater inhibition of ASR than that of R1 may be the consequence of multiple startle reflex inhibition gates. The results of Exp 5 support this notion, showing strong SON-induced LateR inhibition by the SON prepulse because of a more complex pathway than R1 through the spinal trigeminal nucleus in the medulla oblongata and several interneurons (Kimura and Lyon, 1972; Ongerboer de Visser and Kuypers, 1978; Aramideh et al., 1997; Cruccu et al., 2005). Although both the startle reflex pathway and the PPI site remain unclear (Swerdlow et al., 2001), some regions modulating the PPI circuit have been identified, such as the caudal pontine reticular nucleus (Koch and Schnitzler, 1997). However, each sensory system likely has its own inhibitory mechanisms, and each pyramidal cell is controlled by several types of inhibitory interneurons (Karnani et al., 2014). This means that a leading stimulus changes pathway excitability, which modulates the subsequent processes in the pathway. It is probable that complicated PPI pathways show wider ranges of modulation because of multiple steps.

### Clinical implications

Along with reflex pathway normality, circuit excitability can also be examined by PPI (Schicatano et al., 2000). In the present study, signals ascending through the SON work in at least two ways: direct excitatory transmitter release at the nerve terminal; or the indirect production of IPSPs via inhibitory interneurons at the synaptic transmission to the principal nucleus, presynaptically or postsynaptically. The latter controls the timing and intensity of pyramidal cell firing. Figure 6 shows a simple example of a feedforward inhibitory microcircuit. As the prepulse activates similar mechanisms and the pyramidal cell interacts with many interneuron types (Karnani et al., 2014), the excitation/inhibition balance and IPSP time course of the circuit determine the process modulation at each PTI. Therefore, PPI at a certain PTI is expected to resemble the inhibitory function of an interneuron, for example, the somatostatin-positive interneuron for long-latency inhibition (Silberberg and Markram, 2007). There are some diseases that involve inhibitory function deficits, such as schizophrenia (Braff, 2010), epilepsy (Schuler et al., 2001), and developmental disorders (Hussman, 2001; Braat and Kooy, 2015). These deficits may or may not be anatomically and neurophysiologically specific. For example, in autism spectrum disorder, alterations of the GABA system have been reported in various brain regions (Viscidi et al., 2013; Cellot and Cherubini, 2014), suggesting region nonspecific deficits in the inhibitory function. R1 PPI is expected to reflect the function of the canonical inhibitory system and, thus, be a useful measure to elucidate the pathophysiology of such diseases. In a study using guinea pigs, GABA_B_ receptors were involved in paired-pulse suppression of the R1 component (Pellegrini and Evinger, 1995).

In addition to the reflex pathway inhibitory microcircuit, PPI is useful for detecting changes in control from the higher brain regions (Valls-Sole, 2012). A good example of this is the late blink reflex hyperexcitability in Parkinsonism (Kimura, 1973). In Parkinson's disease, there is reduced tonic inhibition from higher brain structures to the trigeminal blink reflex circuit (Basso et al., 1996; Basso and Evinger, 1996); thus, the hyperexcitable circuit exhibits weaker PPI than controls (Nakashima et al., 1993). Other diseases relating to the basal ganglia also show reduced PPI of startle reflexes including blepharospasm (Gómez-Wong et al., 1998), Huntington's disease (Valls-Solé et al., 2004), and Tourette syndrome (Swerdlow, 2013). However, each disease influences the reflex circuit differently, so more than one technique may be necessary to differentiate the responsible mechanism. The prepulse–test combination and its variations are one such candidate.

In conclusion, this study demonstrates the electrophysiological nature of the R1 blink PPI elicited by SON stimulation. Results from five experiments indicate the principal nucleus of the trigeminal nerve to be the target site of R1 PPI. PPI for all of R1, trigeminal late blink reflexes, and the auditory startle reflex showed a similar time course as a function of the PTI, suggesting a set of ubiquitous PPI mechanisms. Thus, the present results are consistent with the conjecture that PPI is not specific to the startle reflex but represents a nonspecific self-regulatory sensory-processing mechanism (Inui et al., 2016). R1 PPI, in conjunction with other paradigms with different prepulse–test combinations, would increase disease understandings that relate to abnormal inhibition. The time course of PPI, as a function of the PTI, may represent several temporally overlapping inhibitory mechanisms (Inui et al., 2016), that is, the summation of several temporally distinct IPSPs. Thus, while R1 PPI is simple in terms of inhibition site, this does not mean that the interpretation is also simple. To better understand the significance of R1 PPI, these components should be identified and separated. The effects of age and gender were not determined because of the small sample size in the present study. Further studies with larger sample sizes are needed, as such effects are known for the PPI of the acoustic startle reflex (Ellwanger et al., 2003; Aasen et al., 2005; Swerdlow et al., 2017) and the trigeminal blink reflex (Kofler et al., 2013).
